# Solidarity, vulnerability and mistrust: how context, information and government affect the lives of women in times of Zika

**DOI:** 10.1186/s12879-020-04987-8

**Published:** 2020-04-03

**Authors:** Ana Rosa Linde-Arias, Maria Roura, Eduardo Siqueira

**Affiliations:** 1 Mauricio Gastón Institute for Latino Community Development and Public Policy, University of Massachusetts Boston, Boston, USA; 2 Graduate Entry Medical School, University of Limerick, Limerick, Ireland; 3 Health Research Institute, University of Limerick, Limerick, Ireland; 4 School for the Environment, University of Massachusetts Boston, Boston, USA

**Keywords:** Zika, Women, Social determinants, Information, Public health, Maternal and child health

## Abstract

**Background:**

The public health response to Zika outbreak has mostly focused on epidemiological surveillance, vector control, and individual level preventative measures. This qualitative study employs a social-ecological framework to examine how macro (historical, legislative, political, socio-economic factors), meso (sources of information, social support, social mobilization) and micro level factors (**i**ndividual actions, behavioral changes) interacted to influence the response and behavior of women with respect to Zika in different contexts.

**Methods:**

A qualitative study was carried out. Women were recruited through the snowball sampling technique from various locations in Brazil, Puerto Rico, and the United States. They were of different nationalities and ethnicities. Data were collected through semi-structured interviews. The data transcripts were analyzed using thematic analysis.

**Results:**

Women in this study deemed the information provided as insufficient, which led them to actively reach out and access a variety of media sources. Social networks played a vital role in sharing information but also resulted in the spread of hoaxes or rumors. Participants in our research perceived socio-economic inequities but focused on how to remedy their microenvironments. They did not engage in major social activities. Lack of trust in governments placed women in vulnerable situations by preventing them to follow the guidance of health authorities. These impacts were also a result of the response tactics of health and government administrations in their failed attempts to ensure the well-being of their countries’ populations.

**Conclusions:**

Our findings call for public health interventions that go beyond individual level behavioral change campaigns, to more comprehensively address the broader meso and macro level factors that influence women’ willingness and possibility to protect themselves.

## Background

In November of 2015, the Brazilian Minister of Health declared the Zika epidemic a Public Health Emergency of National Concern due to the critical increase of microcephaly cases in the Northeast of the country [1]. By February 1st, 2017, 76 countries have reported the presence of Zika. Of the 205,013 cumulative confirmed cases of Zika infection across the world, 130,840 were in Brazil [2], where differences by region and level of education in access to and use of health services persist. In fact, most microcephaly cases were concentrated in Northeastern Brazil, where health inequities are higher.

Although news media outlets are no longer concerned with Zika, transmission continues in 87 countries and the U.S. [3]. Puerto Rico has witnessed the most negative known outcomes of ZIKV infection in the U.S. [4]. Concerns should be raised not only regarding the likelihood of the virus persisting in currently affected areas, but also the danger of its spreading to new areas throughout the globe.

The *Aedes aegypti* mosquito is the principal vector responsible for the widespread transmission of the virus [5]. The ZIKV usually causes only a mild infection in humans, but it is also associated with severe cases of Guillain-Barré syndrome and death. For women who are pregnant it can produce severe neurological complications and adverse fetal outcomes [6]. During pregnancy it can trigger congenital brain abnormalities [7]. Sexual transmission of Zika from both male and female partners can occur [6, 8] and the virus can remain viable in semen for months [9]. These unique adverse effects of Zika for maternal and perinatal health call for a broad spectrum of public health interventions.

Governments and multilateral agencies issued general advice and recommendations to protect women [10]. Most of these messages where focused only on women. Many governments in Zika-affected areas strongly advised women to avoid becoming pregnant [11], whereas others stressed the importance of obtaining counseling and adequate access to family planning resources [12]. However, populations do not have equal access to information on contraception and in a number of countries in the Americas, such as Brazil, the country most affected by the Zika outbreak, abortion is illegal. However, to date the changes in legislation in either country have yet to take shape [13]. Preventive measures also included recommendations such as avoiding locations likely to contain infected mosquitos, covering oneself completely with clothing, and applying repellents, among other individual level preventative measures [14, 15].

The public health response to the Zika outbreak mostly focused on epidemiological surveillance and vector control, together with individual level behavioral change campaigns However, women had to deal with the harrowing threat of mosquito infection and lived in communities exposed to upstream factors such as media, public policies, and political circumstances that determined how the epidemic influenced their daily lives [5,17, 18]. These factors subjected women to a degree of greater or lesser vulnerability. We described elsewhere how the lives of women were deeply affected by the emotional impact caused by the Zika epidemic [16]. However, to have a comprehensive understanding of the impact on women of the ZIKV pandemic, studies have to identify and analyze how the upstream or macro-level factors interact with individual-level maternal and child health outcomes.

During an infectious disease outbreak, particularly one that involves a new or previously unseen health threat, it is important to know how information influences and is perceived by the public. Years of research in communication and psychology show that public opinion change is much more challenging than opinion formation [17]. To be most effective, public health campaigns must provide information that helps the public understand the causes of the disease in media formats that are accessible to various ethnic and socioeconomic segments of the population. Another important factor to consider is how the political contexts and history of a country influence the population reaction to an epidemic such as Zika. Many countries in the Americas where Zika was present had challenging political and economic circumstances. For instance, Brazil, which had the greatest number of Zika cases, is a country with high levels of economic inequality and structural deficiencies in sanitation and health care. Most women and children affected by Zika lived in arduous conditions. As the Zika epidemic evolved, Brazil went through one of the greatest economic and political crises in its history [18]. Puerto Rico, also greatly affected by the epidemic, suffered from socioeconomic inequality and a strong economic crisis. Women in Puerto Rico have historically suffered the effects of forced sterilization [19]. Such legacies are still felt in the current Zika epidemic, since *“effective action has been complicated by lingering suspicions related to historical activities…. Misinformation has clouded… the best ways to protect individuals and communities”* [20].

Women, on whom the responsibility and weight of public policies to control the Zika epidemic rested, must have a role in making informed reproductive decisions, protecting their health, and understanding the implementation of public health policies. The public health response to Zika should consider how women’s organizations interact with the overall society, how they support each other, and whether networks between them do exist. The ability of local communities to mobilize and collaborate for action influences the success of strategies to control infectious disease [21]. Community engagement could be an essential tool to minimize suffering, increase emotional and mental health and support among ZIKV patients and families with affected children.

While broad socio-ecological models have been previously employed to account for the links between individual, community, and broader structural factors, and informed multi-sectorial responses to other epidemics, such as AIDS [22, 23], they have been insufficiently employed to inform the response to the Zika epidemic [24]. In addition, few studies have so far been conducted to address the impacts that the Zika epidemic had on women’s lives at the transnational level, and the ways in which women dealt with Zika outbreaks have yet to be documented. The psychosocial implications of the Zika epidemic are essential for a complete understanding of its long-term repercussions. To our knowledge, few studies discussed the impacts on women who are indirectly affected and less vulnerable to Zika [16, 25]. Women indirectly affected by the epidemic suffered indirect effects from it without being directly exposed to the virus by personal contacts or infected by it.

This qualitative study aimed at furthering our understanding of the impacts of the Zika epidemic on women living in different contexts. Informed by social-ecological frameworks, we identify how macro (historical, political, legislative, socioeconomic factors), meso (sources of information, social support, and social mobilization) and micro level factors (**i**ndividual actions, behavioural changes) interacted to influence the response and behavior of women in different contexts.

## Methods

### Study design

This qualitative study consisted of semi-structured interviews of 34 women who lived in various locations in Brazil, Puerto Rico, and the United States. They were from different countries, socioeconomic strata, religious beliefs, ages, and cultures. They lived in Brazil, the U.S., and Puerto Rico, which have different legal systems, public health policies, and socio-cultural contexts. We chose Brazil and the U.S. as sites for the study because some of us live in Massachusetts and have social networks in Brazil, the continental U.S., and Puerto Rico.

We adopted the snowball sampling technique to recruit participants. Our connections increased ed. trust in the interviewer and facilitated disclosure of personal information. A combination of convenience, snowball, and maximum diversity sampling was thus employed to select out study participants. We recruited women of reproductive age (18–45 years old) living or migrating from countries (such as Brazil, Venezuela, or Colombia) or U.S. states where the ZIKV was detected. The interview guide was pilot-tested and iteratively adapted according to the feedback provided, allowing participants to suggest issues important to them. Details about the guide can be seen in the Additional file 1.

We conducted in-depth interviews with six key female informants, who worked with women directly affected by the ZIKV, to collect expert information on the impacts of the epidemic on such women. All our key informants had significant experience with populations affected by the ZIKV virus in Rio de Janeiro, Brazil; Puerto Rico or the U.S.

### Data collection

All the interviews were conducted by the first author between October 2016 and December 2017 in English, Brazilian Portuguese, and Spanish. We conducted 8 in person interviews and 16 via Skype. The interviews lasted for 2 h on average. They were transcribed verbatim in English, Portuguese, or Spanish by team members who were native speakers of each language. Dr. ARLA, a native speaker of Spanish and fluent in Brazilian Portuguese and English, transcribed all interviews. All interviewers were kept anonymous. Dr. ARLA checked recordings for accuracy.

### Data analysis

**Fig. 1:**
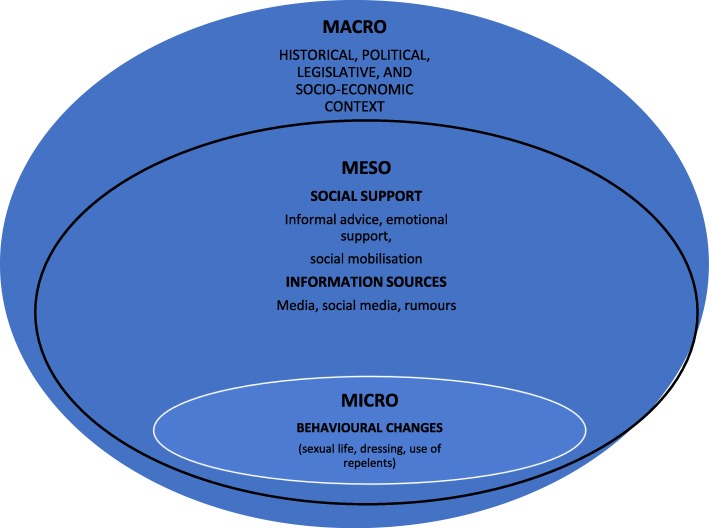
Socio-ecological approach to the ZIKA epidemic

Our data analysis was informed by socio-ecological frameworks [26, 27] and categorized interlinked factors at the macro, meso and micro levels. The macro level consisted of historical, political, legislative, and socioeconomic factors. The meso level included sources of information, social support, and social mobilization. The micro level included **i**ndividual actions, and behavioural changes (protective behaviours). Recurrent topics were assigned codes and sorted into categories and subcategories within an initial coding frame that accounted for the diverse layers of health determinants underscored by socio-ecological theoretical frameworks (Fig. 1). Transcripts were systematically coded by Dr. ARLA using NVivo software® for data analysis. The process began by generating a few free nodes; then ideas or key words derived from the interviews were used to code the text into main themes and derived themes, usually called coding tree. Codes continued to be created, merged, and modified as data collection and analysis progressed, employing a combination of deductive and inductive approaches. Dr. MR and Dr. ES collaborated with Dr. ARLA to create the structure of the node tree. They coded several interviews independently and discussed the node tree during several meetings before consensus was achieved. We adopted pseudonyms for all participants to maintain anonymity.

### Ethical considerations

The research protocol was approved by the Ethics Committee of the Institutional Review Board of the University of Massachusetts Boston under number 2016186. All participants were informed of the study aim and procedures and advised that participation was voluntary and confidential. Written consent was obtained from those who agreed to participate in the study.

## Results

### Micro-level factors

#### Participant characteristics

Thirty-four women, with ages between 22 and 41 years, participated in our study. As seen in Table 1, 20 women were married while 14 had long-term partners. They resided in Florida, Massachusetts, Washington D.C., Puerto Rico, and several localities of Brazil. Participants self-reported their ethnicities as Brazilian, Hispanic, and Americans. We did not collect information about the socio-economic characteristics of participants. We inferred that information by the content of the interview and occupation of participants, who ranged from lower middle to upper middle class. All participants lived in urban areas. Eighteen interviewees had been pregnant recently or were pregnant at the time of the interview, while eight planned o get pregnant and eight did not want to become pregnant though lived in places where Zika transmission occurred. Two participants were misdiagnosed with Zika while pregnant, one had a husband diagnosed with the virus while pregnant, one was suspected of bearing a child with microcephaly, and six had a positive diagnosis of Zika though not pregnant. All interviewees had at least high school education; eight held Doctoral degrees, six held Master’s degrees, six were in postgraduate studies, and ten had college degrees. Participant religious beliefs included Catholicism, Evangelism, Spiritism, Agnosticism, and Atheism.

**Table 1:** Sociodemographic Characteristics of the Women Interviewed

Characteristic	n
**N**	**34**
**Age Range**	
22–30	15 (44%)
31–41	19 (46%)
**Self-defined Ethnicity**	
Brazilian	15 (44%)
Hispanic	14 (41%)
American	5 (15%)
**Civil Status:**	
Married	20 (58%)
Single/ In a Relationship	14 (42%)
**Maternity status / plans**	
Recently born baby	8 (24%)
Pregnant	10 (28%)
Planning to get pregnant	8 (24%)
No plan to get pregnant	8 (24%)
**Residence**	
Brazil	10 (29%)
Washington DC	1 (3%)
Massachusetts	6 (18%)
Florida	7 (21%)
Puerto Rico	10 (29%)

#### Emotional wellbeing

Reported effects of the Zika epidemic at the individual level included reduced physical and emotional well-being, feelings of isolation, sadness, and uneasiness. Interviewees had a strong feeling of uncertainty and mistrust concerning unknown factors surrounding the epidemic, which contributed to helplessness and distress. Fear, panic, concern, angst, and tension were commonly expressed. Maria, from Miami, summarized well her insecurity:

“*So I had the feeling of not knowing what it was. I was protecting myself from a thing I do not know exactly what it is, what do I have to protect myself for? It was a very tense experience…”*

Sometimes, the women voiced feelings of sadness, responsibility, shame, failure, and even guilt because of the pressure of having a healthy child. Pressure to avoid contagion led many women, facing the potential to be infected with Zika, to feel guilty and lonely. Ana C., for example, mentioned that she had feelings of failure due in part to government and media messages that blamed women who got bit by infected mosquitos as not careful enough to protect themselves. Many women expressed living in constant fear or anxiety of having to avoid or prevent mosquito bites from affecting their own health or that of their unborn child. As Katherina B. commented:

*“We were not going to have any other kids after this and so this is really sad that it’s happening here, at this time, when I don’t really get to enjoy it.”*

#### Behavioral changes

The daily routines of women were drastically changed by Zika. In order to protect themselves from the threat of the virus, women adopted behaviors that caused substantial changes in their social lives and personal wellbeing. They often sprayed themselves with chemical repellents throughout the day and wore long dresses and long sleeve shirts despite residing in tropical climates with warm temperatures. A significant adjustment to what should be considered normal routine behavior was described by Mariana S.:

*“[I] bought a repellent and developed a routine that I put it on every time I showered. I used to put it on as if it were cream… like brushing your teeth and I would put it on every time I went outside. I tried to wear long clothes and not open shoes. I bought a product that I never used, to put on [my] clothes. [It] was very strong because, at the same time, these are chemicals to protect against Zika, but I’m also pregnant with a lot of chemicals all day long…”*

The daily lives of study participants, as reflected in their social relationships and interactions with their partners, family members and children, were greatly affected by Zika. Their intimate relationships with partners suffered both emotionally and sexually, and the fear that Zika could be contracted via sexual contact caused a strain in the relationship. Women often reported feeling isolated from their partners, children, parents, relatives and extended families. Lack of leisure, social, and outdoor activities, also contributed to social isolation. Reports of disruption in their social lives and daily routines were common. Participant Marilyn G. details her personal experience:

*“…It affected [the relationship] because [Zika] is a stress. You are worried, all the time if there is or isn’t a mosquito [present]… a constant focus of tension, the quality of life falls a lot because that affects the relationship. I had places that I would not go to… because I thought there could be mosquitoes.”*

At the professional level, women placed their careers at risk by giving up growth opportunities such as attending meetings and job-related trips. Effects on the sexual and reproductive life included renouncing pregnancy or postponing their decision to motherhood, and in a few cases sexual abstinence as a form of protection.

### Meso-level factors

#### Social support

Women with varying levels of contact with the ZIKV showed solidarity. Testimonies of empathy, concern, as well as support for one another, indicate continuous conversations about the effects of the virus. Throughout the Zika epidemic, participants came to see themselves in other women who could potentially be infected by the virus, as Interviewee 1, from Brazil, stated:

*“…here we only talk about it. [In Brazil] everyone talks about it. It has a climate, a very strange atmosphere. You cannot look at a pregnant woman on the street and not think, not imagine…”*

Communitarian feelings, particularly among pregnant women, flourished because a sense of commonality developed due to fear of the adverse effects caused by Zika. The risks of Zika created a climate of sincerity among women to discuss scientific uncertainties, potential methods of prevention, and to provide informal counseling based on personal experiences. The unfounded or inadequate support from the broader community was replaced by the unity among women that had initially limited relationships with one another. Respondents demonstrated empathy with each other:

*“…I saw people in solidarity with pregnant [women]… like [a young] girl at work…. Everyone worried about her. During high heat [temperatures] people worried… because she was all dressed.... They always asked her if she was okay. And she was pregnant with Zika before she knew she had it. She got Zika, became pregnant a few months later, and the baby was born normal.”* (Interviewee 2, Brazilian).

Yet, many participants felt that these support networks were small, stemming from individual initiatives. This mutual solidarity happened to a degree in already established women networks, but there was lack of support from government agencies. In addition, the epidemic did not encourage women’s political or social activism. Many women were concerned only with their own well-being or that of people close to them. They did not step outside of themselves to mobilize and change women’s health, as Interviewee 3, from Brazil noted:

*“Everybody remained more worried about their own micro-environment, what I can do to protect myself, what I can do to protect my future baby so that it does not have microcephaly. Very few joint actions, from women to women to women.”*

There were contrasting experiences regarding stigma depending on where they resided. Participants such as Isabel from South America indicated no such stigma when discussing Zika infections:

*“No, I do not see it like that, not like a social stigma. It is not only Zika that affects the population or classes…. The truth is public health policy, the lack of sanitation and hygiene; there are some favelas that remain near the river. There, they have mosquitoes in those places. They are more exposed.”*

Conversely, women residing in the United States pointed out that stigma towards the ZIKV was, in fact, prevalent. In particular, if individuals were from Central or South America, there were questions about whether they could potentially be carriers of the virus. Interviewee 4 from Miami said:

“*At the airport I felt kind of intimidated by big signs asking: do you come from South America?”*

And discrimination played a part in feelings of stigma. Some women indicated feeling targeted for their ethnic background, as Interviewee 5 from Miami described:

*“There are so many Latinos in Miami that they even brought Zika…”*

In sum, while there was equality among the women when discussing their experiences with the Zika epidemic, there were perceived inequities in its outcomes.

#### Sources of information

From news programs on television to a variety of online platforms - wherever or however the information could be retrieved- participants consumed information from numerous media sources as the cases of Zika infections increased. Some, like Interviewee 6 from Puerto Rico, concentrated on accessing sources of information that they considered dependable or that fit into their daily life needs:

*“As I use the internet a lot, I see more news on the internet. The newspapers, I read a lot of newspapers on the internet. I was educated enough with that, but television I do not see much. I am more into computers.”*

Social media played an important role in not only providing access and sharing of resources among women’s networks, but also information on how the virus spread. Much of the information available on such media outlets led many to misperceive it as concrete, well-defined and accurate facts. Individuals in different countries in the Americas acquired similar knowledge and perceptions on how to avoid Zika from similar media sources. However, in some locations the information available was limited, especially if mosquito-borne viruses were not usual, as in the United States. For example, Interviewee 7, from the U.S., affirmed:

*“It seemed to me that here in America, as they did not have as much experience, they did not have so much data... that the biggest information was from cases in Brazil that were like further from having implications to the U.S., etc...”*

The Zika epidemic was recognized as a media boom by all women interviewed. According to them, the presence of ZIKV almost immediately took over the airwaves, as Valeria from Brazil summarized: “*It was like a boom that came out.”* Nonetheless, just as quickly as the virus became known, the media boom was suddenly over and a complete disappearance of Zika news in the media ensued. Interviewee 8, from Brazil, recalled:

*“Today I don’t see that anymore… for example in Rio. If you were to look at the TV campaigns or any of those things, it disappeared. It’s only going to re-appear during the summer.”*

Much of the informational material disseminated during the outbreak of Zika focused primarily on prevention methods not based in scientific assessment of the epidemic; there was limited access to scientific information with a more comprehensive approach to the disease. In fact, many participants, such as Interviewee 9, from Brazil, viewed the evidence available to the public as incomplete:

*“One also looks for what one needs, tranquility, but what I think is that there was not much advanced scientific information, studies that really gave information about this. It was more focused on prevention.”*

A number of women reported encountering alarmist materials as the epidemic expanded. Many rumors appeared, causing quite dangerous levels of chaos and mistrust. Participants recalled speculations that included rumors such as *“Zika is a government invention”* or *“Monsanto introduced the mosquito.”* Thus, women felt trapped in the rumor mill that Zika produced. Many were unsure about where to turn for reliable information on the virus or wondered if the sources available were trustworthy.

Access to social media also solidified the ability of individuals or groups to spread false rumors, detrimental and harmful to women’s health. Personal opinions became concrete facts leading many not to comprehend fully the severity of Zika along with the true consequences of infection, as Interviewee 10, from the U.S., claims:

*“…they are telling other women in those groups ‘It’s not a big deal’ and I am like ‘You are not a medical doctor.’ Other groups that I am part of, they are very much concerned about it, and they try to help each... send articles to a few moms that were pregnant for them to be aware.”*

As a result of the rumors and unsubstantiated information, a sense of suspicion developed towards the news or updates that appeared in social media. Furthermore, there was much confusion as to whether the virus was truly detrimental and dangerous or if the media stories were gossip or “fake news.”

### Macro-level factors

#### Distrust of governments

The perceived role of governments and public health organizations, and their past responses to citizens, is a major factor in explaining the negative attitudes of women toward the Zika epidemic. Participants argued that the public health system placed the responsibility of preventing any type of health complications from Zika onto women who had limited abilities to eradicate mosquitoes. Many women stated that these measures were invasive, while creating the perception that they were the sole determinant of whether or not they contracted Zika. Their perception was that public health officials focused on eradicating the vector (mosquito) and on preventing microcephaly, both of which placed the burden of prevention on women.

In the case of Puerto Rico, women reported an adversarial relationship between the government and the population, even in the face of an extreme health emergency. The political history of the island seemed to shape the attitude of women regarding the Zika epidemic, an example of which is seen in the words of Interviewee 11:

*“[People are] skeptics, because in Puerto Rico the feds are always saying things to scare people and they have used Puerto Rico as guinea pigs for a lot of clinical trials... People do not trust any medical related issue from the government.”*

Past political tensions continue to mold the lack of trust of Puerto Ricans in government recommendations regarding public health. There was a distinct sense of vulnerability among Puerto Rican participants since they could not trust official government information, which was evident when Zika landed in the island. According to Interviewee 12:

*“Somehow, in truth, the government did not want Puerto Rican women to get pregnant. I do not know. It may or may not be [true]. In truth, I do not explain myself well, but I feel it [Zika] was an exaggeration.”*

Some Brazilian participants also viewed Zika with skepticism and felt a sense of resignation towards the government’s responses to control the virus. They indicated that during past mosquito-borne epidemics the measures taken to combat the diseases were similar in nature, and the newly identified virus would not bring about any difference due to a supposed sense of normality within chaos. Interviewee 3 makes this view clear:

*“…it’s like violence in Brazil. You read about it and you get afraid. Coming back is like you adapt to that. It’s not too scary, you are not apprehensive all the time. It’s like with my friend. We were worried but when you come here, you are back to your routine.”*

#### Inequities among women regarding Zika

Despite genuine gender solidarity for one another -given the possibility that malformations and developmental disabilities could occur - there remained ingrained elitism and social hierarchy in the views held by some women. Nevertheless, many participants clearly understood that the mosquito was a great equalizer. They realized that despite their relatively privileged socio-economic background, women from all backgrounds could be equally exposed, to a certain extent, to the virus. In that sense there was equality. But there was no equality in combating the epidemic. Essentially, the women understood that,

*“…a mosquito bites anyone the same…. It does not understand what is rich or poor”* (Interviewee14, Puerto Rico).

Some participants were quite aware of their privilege. They felt some guilt for their higher socioeconomic positions, which allowed them more access to information and better understanding of Zika. Although the ZIKV was an “equal opportunity virus,” socio-economic factor dictated the lives of women during the epidemic. Interviewee 15 from Miami pointed out clear indicators of privilege, even when accessing informational materials on Zika:

*“I could use my network of colleagues and scientists to find out even more information like anything new that hadn’t necessarily been published yet… I really feel like I was lucky that I could do that.”*

Participants from financially stable and affluent situations easily identified barriers facing poorer women that could create further challenges in their attempts to protect themselves against Zika, but social solidarity that would push for changes in women’s health rights or economic stability as a whole were not their major concerns.

## Discussion

Individuals’ responses to the epidemic depend on the sources of information received, and how these are perceived, which is in turn influenced by broader political, historical, legislative and socioeconomic contexts (Fig. 1). Few studies in the literature [28–31] address the experiences of women indirectly affected and less vulnerable to the effects of the Zika epidemic. Our study demonstrate that the social effects of the epidemic affect more women than had been thought before and at deeper levels. They are coping with feelings of fear, helplessness, and uncertainty by taking drastic precautions to avoid infection that affect all areas of their lives. Coping strategies pose obstacles in professional life, lead to social isolation, including from family and partner, and threaten the emotional and physical well-being of women [16].

The first step in responding to any health epidemic involves having adequate information on the causes, how the disease may be contracted, and what steps should be taken to ensure one’s safety and health. Dissemination of information plays an important role in epidemics, especially with those comparable to Zika, where all aspects of it were new and there was ignorance among citizens and health professionals alike [32]. Legacy and social media respectively influence risk perceptions and protective behaviors during emerging health threats [33].

During the Zika outbreak, women in our study deemed the information provided by public health officials as insufficient, which led them to actively reach out and access many media sources to counterbalance information gaps and shortcomings. Social networks played a vital role in sharing information but also resulted in the spread of hoaxes or rumors. It has been reported that Public Information Officers who were monitoring social media felt better prepared for Zika and were more satisfied with their crisis management since social media facilitates the spread of both accurate and false information [34]. Understanding these media effects is essential to communicate public health information and engage different populations in the community [35]. Above all, the need to access social media to gain knowledge about the debilitating virus clearly demonstrates the lack of satisfaction with then available official information about Zika.

There was a banalization of Zika by the average women and also health professionals, who were unclear about the health effects of the virus. When a disease is endemic in a particular population, reactions tend to be much more subdued, and it is harder to induce protective behavior [36]. As an example, while international media attention has focused squarely on the risks posed by the Zika epidemic— prompting extreme reactions in some cases, such as Olympic athletes pulling out of the Rio games— evidence suggest that Brazilian residents may have viewed it differently, using the frame of reference of a familiar endemic disease, dengue, to evaluate the risk of the new and unknown Zika [37]. Several studies of dengue in the developing world have underscored the difficulties of stimulating a strong public response or sustaining one after that epidemic subsided [38, 39].

In the case of Puerto Rico, the history of government sanctioned medical trials [40, 41] and forced sterilization [42] caused the population to become vulnerable, because it perceived current and future health epidemics as unpredictable. Such effects are so long-lasting that even in the current Zika epidemic, segments of the population not only doubted government intentions, but also refused to adopt any advice provided by the Department of Public Health. The voices of participants suggested that there are individuals and populations that resist the idea that government can truly be sympathetic to their well-being, whether the issue at hand is health, safety, or security. In fact, Zika brought to the forefront the interpretation that government abuses on a given population can have long-term harmful consequences.

The interaction between women and society as a whole is crucial to understand how stigma, classism and gendered inequalities happened during the Zika epidemic. We focused on how women perceived stigma, since during past epidemics, a negative social attitude had been reported [43, 44]. In our case, it became clear that the mosquito was perceived as an equalizer; the mosquito does not discriminate between rich and poor, biting everyone equally. Quite a different reaction was experienced in other epidemics, such as Ebola, where a definitive association of the disease with only “poor people” was patent [45], or in the case of HIV where stigma has persisted even after provision of effective treatment [22].

Given the social inequities in the U.S. and Brazil, one would assume that social stigma surrounding Zika would be prevalent; however, there were contrasting experiences among women depending on their geographic location. Brazilian women showed no such stigma about Zika infections. Conversely, U.S. women argued that stigma towards the ZIKV was, in fact, prevalent. This lack of stigma could be explained by the fact that Brazilian women are more accustomed and knowledgeable about these kind of mosquito transmitted diseases, such as dengue, chikungunya, which are rather common where they live.

Discrimination played a part in feelings of stigma whereby women felt targeted for their ethnic background. The U.S. government claimed that Zika was associated with immigrants from Central and South America, after Zika was recognized as a public health emergency in the U.S. The blame for the virus infection was again placed on individuals from a few nationalities as opposed to mosquitoes.

The Ebola infection also had similar impacts. Most of the individuals infected resided in poorer nations in the African continent. Women in these areas were the most affected as they had inferior access to healthcare [44, 45]. Information was either limited or the sources of knowledge were not culturally competent enough to be shared with the population. Although the virus could indeed be contracted via exchange of bodily fluids from person to person, individuals were charged with the responsibility to maintaining a clean bill of health [46].

Participants in our study understood that anybody could be bitten by the mosquito, but most were aware that they were privileged while others were in much more vulnerable situation. They clearly perceived the ethnic and socioeconomic inequities deeply rooted in their different contexts. However, they mainly focused on how to remedy their microenvironments.

Social movements to support and defend women’s reproductive health rights because of the Zika epidemic might have happened, but many women did not realize that the connection they were fostering with each another might have evolved into a massive push for better access to birth control and better health care services. Surprisingly, women’s activism in Brazil and other Latin American countries revolved around the creation of an underground movement for health care - women with children affected by Zika formed non-profit organizations and informal networks to defend their rights [47, 48]; abortion activists carried out many activities and studies [2, 49, 50] in countries where abortion was illegal [29].

Some women resisted but a movement for real change was invisible, maybe because advocacy efforts were top-down and thus unsuccessful at generating grassroots mobilization. The views of advocates might not be aligned with women’s’ views regarding abortion, for example. In a previous transnational study that provided insights into women’s views and attitudes towards their reproductive rights in times of the Zika epidemic, we reported that reproductive decisions were intimately related to personal convictions and cultural beliefs, and their actions and thoughts were embedded in their sociocultural norms [25]. Thus, it is important for the advocacy to be culturally sensitive, so it reaches a broad spectrum of the population.

The lack of social mobilization seriously addressing Zika as a global health matter is the biggest difference with other global epidemics. In the case of HIV there were huge mobilizations to get free treatment for all [51], tackle stigma [52] and to investigate women controlled prevention measures such as microbicides [53]. Also, since Zika has similar symptoms to dengue and chikungunya, some participants of our study reported feelings of resignation to the ongoing epidemics. Another factor that might have contributed to the lack of massive social response is the lack of support for women’s reproductive health and rights that have historically been neglected aspects of Public Health [54, 55].

Political contexts also affected the social response, as reported by women in Puerto Rico and Brazil. In the end, the responses ended up becoming for the most part individual efforts to manage their own microenvironments. It is clear that the women more affected by the ZIKV infection, are the poor ones, traditionally neglected in their living conditions and health [56]; but our study shows that all women were affected in some way. We indicate that the social impacts of the epidemic affected more people, directly and indirectly, than had previously been thought and at deeper levels. Zika is a vast and far-reaching epidemic that altered the lives of women of all social positions.

Regardless of the downgrade issued by the World Health Organization in November 2016, there are many other non-medical impacts of the ZIKV. Those impacts derived from the failed or insufficient response of health and government administrations to ensure population wellbeing by focusing almost exclusively on biomedical approaches. As the case of HIV has shown [57], a broader socio-ecological approach must be adopted for successful implementation of public health policies to control an epidemic.

Our qualitative research is limited by the small number of participants, which does not allow for statistical generalization. However, it is conceptually generalizable in the sense that the themes that emerged are relevant to analyze and guide the response to the epidemic in different contexts. This is study analyzed the impact of the Zika epidemic on women who were indirectly and/or directly affected by it and considered the particularities of different contexts.

The strategies to canyontrol Zika in different communities should vary depending on their assets, vulnerabilities, and public health environments. On the one hand, the epidemic may stress public health systems and highlight weaker points that need comprehensive improvements. On the other, the socioeconomic, cultural, and political determinants of the epidemic may also have some bearing on the successes or setbacks of the emergency response to Zika.

An epidemic is a social phenomenon as much as a biological one. Thus, understanding people’s behaviors and fears, their cultural norms and values, and their political and economic realities is essential. Having social scientists and academics working alongside governments and public health authorities would contribute to the introduction of crucial media messages, policies and guidelines to support the affected population. We hope that our work will lead to new guidelines and policies to ensure that the emergency response and the messages are delivered in the most effective way.

## Conclusion

The Zika epidemic demonstrates that women’s health still faces a variety of barriers at the global level. The results of this pilot study suggest the importance of considering social science approaches to understand risk and adopt public health measures to control epidemics. Financial, social, religious and cultural aspects are always involved in epidemics*.* The Zika epidemic was yet another lost opportunity to increase culturally sensitive family planning services, since the particular costs of the ZIKV for maternal and perinatal health demanded a broad spectrum of health interventions. Our findings call for public health interventions that go beyond individual level behavioral change campaigns, to more comprehensively address the broader meso and macro level factors that influence women’s’ willingness and agency to protect themselves.

## Supplementary information

**Additional file 1.** Interview Guide developed by the researchers for the interviews. It included topics such as women’s personal and family life, perceptions and knowledge of Zika, views on reproductive health and rights related to the Zika syndrome.

## Data Availability

The datasets generated and/or analyzed during the current study are not publicly available due to the protection of individual privacy of participants but may be made available from the corresponding author on reasonable request.

## Abbreviations

AIDS: Acquired immunodeficiency syndrome
WHO: World Health Organization
ZIKV: Zika virus
