# Supplementary material for: Solidarity, vulnerability and mistrust: how context, information and government affect the lives of women in times of Zika
Source: BMC Infect Dis. 2020 Apr 3;20:263. doi: 10.1186/s12879-020-04987-8 (PMC7118940; doi:10.1186/s12879-020-04987-8)
Supplement: Supplementary file 1 — Additional file 1. Interview Guide developed by the researchers for the interviews. It included topics such as women’s personal and family life, perceptions and knowledge of Zika, views on reproductive health and rights related to the Zika syndrome. [file 12879_2020_4987_MOESM1_ESM.docx]

**INTERVIEW GUIDE**

Personal data:

We would inquire the following aspects:

Name, Age, Place of Birth, Education, Occupation, Religion, Civil/Relationship Status, Children, Place of Current Residence and time length there, Frequency, location and type of travels.

Topic 1. To identify women’s social and health literacy about Zika.

What do you know about the Zika?

What kind of information do you have? How did you get this information?

Have you talked to health care professionals about Zika? How was it? Did you feel comfortable talking about it with them? Did you get the necessary information from them?

What kind of information was more clear and convenient to you?

Did you feel better after reading the information and/or talking to the Health Care Professionals? Which way has provided the best information to you?

Do you have any doubts about Zika?

What aspect of the Zika epidemic is causing you concern?

What information about Zika is affecting you more? How? Why?

Is it clear to you how to prevent and protect you from Zika?

Topic 2. To determine women’s perceptions and knowledge about the potential and real effects of Zika on their personal and family life.

Do you have a partner? Do you live with your partner? Do you have sexual relationships with your partner? Has the Zika affected your relationship with your partner? How?

Has it affected your sexual life and relationships in general? How?

Are you planning to have children? When? How?

Have you taken any decision concerning your personal, family and professional life because of Zika? What kind? How do you feel about this?

Have you renounce to anything related to your personal, family and professional life because of Zika? What? How do you feel about this?

Do you think that Zika has affected your personal, family and professional life? How?

Topic 3. To characterize the perception and knowledge that women have on their reproductive rights and how government policies influence them.

Do you know the measures/recommendations that are given to prevent the effects of Zika?

Do you know the measures/recommendations related with sexual life? Which ones are they? What do you think about them?

Do you know who dictate these measures/recommendations? What do you think about them? Do they make you feel safe, comfortable? Do you trust them?

What do you think about the Organizations and people that established these measures/recommendations? Do you trust them? Do you think that they are reliable?

Do you understand these measures/recommendations? Do they make you feel safe, comfortable? Do you think that they are reliable? Would you follow them?

Which measures/recommendations are more important? Why?

Do you feel comfortable about these measures/recommendations?

Would these measures/recommendations affect your personal/sexual life? How? What do you think and how do you feel about that? Is that OK with you?

Do you think it is OK that other people dictate these measures/recommendations that could affect your personal and sexual life? How do you feel about this?

Do you think they are interfering with your life? Which measures/recommendations do you think are the ones that could interfere more?

What would you do about it? Do you think they have the right to do this?

Topic 4. To understand how medical care of Zika disease influence decisions women take regarding their life and their reproduction.

Have you visited any region with Zika? What measures have you taken when going there?

Have you contracted Zika? How did you find out? Symptoms, etc. Did you have medical attention? Type, treatment, and recommendations received. How did you feel about then?

Have you received recommendations by health care professionals? Which ones? How was your interaction with the health care professionals? Were they kind? Did you feel comforted? Did you trust them? Which other sources of information have you consulted? Were they useful?

Are you aware of the recommendations for women and reproduction regarding Zika? Have you received them personally? Which ones?

Are you pregnant? Have you discussed Zika with the health care professionals? Which treatment/advise have you receive? Did you get sufficient information? Have you made any decision about your pregnancy? Have you tried to find different alternatives? Do you think you would need extra support? Which one? How? How do you feel about your pregnancy?

If you are not pregnant, are you planning to become in the next future? Is the Zika epidemy affecting your decision? What do you think you would do if you were pregnant now? What kind of actions you think you would take?
